# Unveiling the ABCs: Identifying India's Healthcare Service Gaps

**DOI:** 10.7759/cureus.42398

**Published:** 2023-07-24

**Authors:** Nistara Singh Chawla

**Affiliations:** 1 Neurology, Christian Medical College and Hospital, Ludhiana, IND

**Keywords:** healthcare policy and management, health services accessibility, barriers-to-care, healthcare systems, healthcare inequality, india's healthcare system

## Abstract

The diverse population of India has challenges with receiving comprehensive and accessible healthcare. The shortcomings of India's healthcare system are highlighted in this editorial by looking at the important topics of accessibility, patient and practitioner behaviors, and clinical governance difficulties. Regional differences, inadequate infrastructure, a lack of qualified workers, and cultural issues all have an impact on how easily accessible healthcare is in India. Gender norms, social shame, religious views, and language problems can all have an impact on how people seek healthcare, functioning as barriers to access. In India, clinical governance is challenged by a disjointed healthcare system and insufficient regulatory frameworks.

To address these barriers, it is crucial to enhance healthcare infrastructure, strengthen regulatory mechanisms, promote a culture of quality improvement, provide training on clinical governance, and leverage technology for data collection and analysis. To reduce gaps in culture and promote access to healthcare, collaborations with local organizations, religious institutions, and community leaders are crucial. In India, efforts are being made to increase access to healthcare through programs like infrastructure development, the expansion of the healthcare workforce, health insurance coverage, and telemedicine. To improve the availability, affordability, and caliber of healthcare services, sustained efforts are required. To reduce the gaps and attain universal and equitable healthcare in India, a complex strategy comprising policy interventions, investments, reforms, and community engagement is required.

## Editorial

India confronts considerable difficulties in providing all of its citizens with comprehensive and easily accessible healthcare due to its large population and heterogeneous healthcare system. Despite impressive development in several areas, India's healthcare system still poses a number of barriers to the patient population [1,2]. A safe, patient-centered, clinically cost-effective, and egalitarian system with the promise of constant and unrelenting improvement makes up quality healthcare. Some of the key factors determining quality are improved outcomes for patients and their caregivers as well as patient satisfaction. Doctors, nurses, staff, paramedics, government agencies, policymakers, and community members are just a few of those who play a significant role in providing, maintaining, and improving quality care [3].

Even after decades of autonomous planning and implementation, the majority of people in developing countries still don't live up to the utopian goal of universal health. In order to present alternate possibilities, this editorial objectively examines the problems and the situation in India. The author highlights three crucial areas: accessibility of services, behaviors of the patients and practitioners, and clinical governance issues, with the goal of illuminating the significant inadequacies in India's healthcare system and offering potential solutions to address these shortcomings.

Accessibility

In India, access to healthcare is a complex issue that is influenced by a number of factors, such as regional inequalities, socioeconomic circumstances, infrastructure constraints, and cultural considerations [4]. Since 80% of experts reside in metropolitan regions, it is noticed that 70% of the population lacks access to such care. Rural residents have only 13% access to primary health centers, 33% to subcenters, and 9.6% to hospitals. Government spending in India is unusually small, with a disproportionate focus on private health spending. The country has a three-tier system of public health care centers in villages, district hospitals, and tertiary care hospitals. In turn, this creates a gap in the availability of subsidies between the rich and the poor in urban and rural areas, with the poorest receiving a small portion while the richest receive more than a third. Both local Indian public health and global public health are affected by this worldview. Even while India has made great strides in recent years to increase access to healthcare, problems still exist, especially in rural and economically underdeveloped areas [5]. Here are some critical elements relating to access to healthcare in India:

Infrastructure

In general, urban areas have more refined healthcare infrastructure, including state-of-the-art medical centers, clinics, and specialty healthcare facilities. On the contrary, a lack of healthcare facilities in rural areas frequently restricts access to healthcare services. Rural India lacks enough infrastructure and poor transportation options, which make it challenging to reach medical services. This results in inadequate medical treatment being provided [6].

Workforce

India being a low and middle-income country also faces a shortage in the healthcare workforce. A barrier to expanding access to healthcare services in India is the distribution and retention of biomedical doctors at public hospitals in rural areas. There is a great disparity in the number of patients and qualified healthcare professionals available to provide services to them. This leads to an overburden of the existing workforce, causing physician burnout [4,7].

Affordability

Healthcare services in India are greatly dependent on the out-of-pocket model. Even though India has the largest private health industry in the world, only one-fifth of healthcare spending is covered by the government; the majority of costs are borne by the patient. About 70% of Indians reside in rural areas and earn a bare minimum daily wage, the majority of which is spent on food and housing rather than health care. Due to the lack of proper government-facilitated health security and insurance schemes, a major chunk of the population is unable to access these facilities due to financial constraints [7].

Cultural Factors

Cultural values, customs, and a lack of health literacy may have an impact on how people seek medical care. Some people could rely on conventional or alternative medicine, put off seeking medical attention, or harbor misconceptions about current medical procedures [4].

Beliefs and behavior

In India, cultural beliefs and practices might, in fact, act as barriers to access to healthcare. People's perceptions, beliefs, and behaviors in relation to obtaining healthcare might be influenced by the diversity of cultural practices and beliefs found in different communities and regions of India. Health awareness campaigns can increase the awareness of people in the rural parts of the country. However, the need of the hour is building capacity, training informal caregivers and volunteers in the community to overcome the stigma [6]. The following are some examples of how cultural considerations affect access to healthcare in India:

Gender Norms and Inequality

Access to healthcare can be impacted by gender stereotypes and inequalities, especially for women. Cultural traditions may restrict women's ability to make health-related decisions, including delays in obtaining care or dependency on male family members' approval. In addition, discrimination and violence against women based on their gender can make it more difficult for them to get medical care. For instance, women and elderly patients frequently need support from family members to get to medical facilities, and they frequently need to get permission before making expenditures on care, getting lab work done, or even going to follow-up appointments [8]. In underdeveloped metropolitan areas, women are confined to their homes and have little freedom to access medical treatment. In order to get medical treatment, they must bargain not only with their husbands but also with their mothers-in-law [8].

Social Stigma

In Indian society, some health illnesses, such as mental health problems, STDs, or reproductive health issues, may be associated with considerable social stigma. Individuals may refrain from getting proper healthcare services or speaking honestly about their health concerns out of fear of discrimination or social isolation. Stigma is especially egregious in healthcare settings, where it has a detrimental impact on patients who are seeking care when they are most vulnerable. The effects of stigma in healthcare settings have been well-documented and range from overt refusal of care, subpar care, and physical and verbal abuse, to more covert actions like having some persons wait longer or handing over their care to less experienced staff members. Therefore, stigma prevents people from receiving the care they need when they are looking for support to maintain a good quality of life, treatment for acute or chronic diseases, or services for disease prevention. It is essential to combat stigma through community awareness and education [9].

Religious Beliefs

In India, decisions about healthcare are greatly affected by religious beliefs. For instance, some religious groups have particular dietary restrictions that could limit their access to a healthy diet. A majority of the Indian population follows vegetarianism due to religious beliefs, which are generally deficient in proteins unless a proper diet pattern is followed. This is one instance where religion and beliefs affect the nutrition of the population. Additionally, there are situations when religious beliefs collide with specific medical procedures, causing people to seek out alternative therapies or refuse all medical care.

Language and Communication

India is a country with many different regional languages. Language barriers frequently make it difficult for patients and healthcare professionals to communicate effectively, which can lower the standard of care. Low literacy rates and lack of awareness create a gap in communication among patients and healthcare providers in India. For instance, in conditions that are palliated, caregivers are often not counseled about dealing with the prognosis due to a lack of better communication.

Clinical governance

It could be described as the framework by which healthcare institutions are held responsible for maintaining proper standards of care and constantly enhancing the quality of their services. Despite what policymakers claim, their budget allocation does not reflect that health is a top concern. This needs to be improved either by ongoing interaction with lawmakers or by incorporating these stakeholders through the establishment of interdisciplinary regulatory bodies [10]. When effectively implemented, clinical governance is meant to increase patient safety, raise the standard of care, and guarantee accountability within healthcare institutions. The successful adoption of clinical governance practices, however, may be hampered in India by a number of obstacles and constraints. The following are some elements that make clinical governance in India difficult to implement:

Fragmented Healthcare System

The healthcare system in India is characterized by fragmentation, with a mixture of independent public and private healthcare providers. This dispersion can make it difficult to develop uniform protocols and recommendations by impeding the coordination and standardization of clinical governance practices across the nation.

Limited Regulatory Framework

Although India has regulatory organizations like the Medical Council of India (MCI) and the National Accreditation Board for Hospitals and Healthcare Providers (NABH), a more powerful and thorough regulatory structure is still required to guarantee compliance with clinical governance norms.

The way forward

In order to address the burgeoning concern about the discrepancies in healthcare delivery systems in India, the outdated healthcare system needs a technological boost. It is essential to enhance regulatory systems and ensure that clinical governance norms are followed. Open communication, patient safety, and a culture of quality improvement within healthcare organizations should be encouraged. Healthcare workers should be provided with continual education and training on clinical governance ideas and practices. The use of digital health tools and technology to help with data gathering, analysis, and clinical outcome monitoring should be encouraged.

Increasing societal understanding of and participation in hospital governance and quality-improvement procedures also holds importance. Healthcare professionals must receive training to comprehend and respect various cultural practices and beliefs. Cultural norms and beliefs should be taken into consideration when designing health education programs, with a focus on the significance of getting prompt and adequate medical care. Collaboration with local organizations, religious institutions, and community leaders can help close cultural gaps and advance universal access to healthcare in India. Governance issues and fragmentation both inside and outside of the health sector have a detrimental impact on the adoption of a coordinated policy strategy for the retention and distribution of doctors in rural areas. It is necessary to implement more policies that address the main issues brought up by stakeholders, as well as better procedures for coordination, accountability, and transparency.

Efforts are underway to improve healthcare access in India. The government has initiated various programs to strengthen healthcare infrastructure, increase the healthcare workforce, and expand health insurance coverage. Public-private partnerships are also being encouraged to bridge gaps in healthcare delivery. Additionally, telemedicine and mobile health initiatives are being implemented to reach remote areas and provide access to healthcare services. However, addressing the challenges of healthcare access requires sustained efforts in enhancing infrastructure, improving healthcare workforce distribution, promoting health insurance coverage, and raising awareness about the importance of timely and appropriate healthcare-seeking behavior.

Conclusion

A multifaceted strategy comprising policy interventions, greater investments, and thorough reforms is needed to close these gaps. In order to increase accessibility, the healthcare system must be expanded, particularly in underprivileged and rural areas, and more healthcare workers must be hired and trained. Reduced out-of-pocket costs and the adoption of universal health coverage programs are necessary to improve affordability. Strict rules, standardized procedures, and expenditures on infrastructure and technology are necessary to improve quality.

In addition, telemedicine and the use of digital technology can assist in bridging the accessibility gap between urban and rural locations. Community involvement, awareness campaigns, and public-private collaborations are also essential for fostering change. India can go closer to realizing its objective of offering fair and complete healthcare services to all of its residents by recognizing and addressing the ABCs of healthcare service gaps - Accessibility, Behaviour, and Clinical governance. The country can effectively close these gaps and guarantee its people a healthier and more affluent future via concerted efforts and cooperative initiatives.
